# Graphene Flakes Decorated with Dispersed Gold Nanoparticles as Nanomaterial Layer for ISEs

**DOI:** 10.3390/membranes11070548

**Published:** 2021-07-20

**Authors:** Barbara Niemiec, Nikola Lenar, Robert Piech, Krzysztof Skupień, Beata Paczosa-Bator

**Affiliations:** 1 Faculty of Materials Science and Ceramics, AGH University of Science and Technology, Mickiewicza 30, PL-30059 Krakow, Poland; bniemiec@agh.edu.pl (B.N.); nlenar@agh.edu.pl (N.L.); rpiech@agh.edu.pl (R.P.); 2 3D-Nano, ul. Lipowa 3, 30-702 Kraków, Poland; kskupien@3d-nano.com

**Keywords:** graphene, gold nanoparticles, potentiometric sensor, hydrophobic material, high electrical capacity, potassium determination

## Abstract

This paper proposes a new type of solid-contact layer based on graphene/gold nanoparticles for ion-selective electrodes. A novel approach to preparing the material for intermediate layer by modifying the graphene flakes by gold nanoparticles is presented. With this approach, we observed a large surface area of material and in consequence high electrical capacitance of electrodes. We have obtained satisfactory results demonstrating that the modification of graphene with gold allows for enhancing electrical and wetting properties of carbon nanomaterial. Electrical capacitance of designed nanocomposite-contacted electrode equals to approximately 280 µF, which in consequence ensures great long-term potential stability defined by the potential drift of 36 μV/h. The modification of graphene with nanoparticles completely changed its wetting properties, as the designed material turned out to be hydrophobic with a water contact angle of 115°. Graphene/gold nanoparticles–contacted electrodes are insensitive to the changing light conditions, exhibiting near-Nernstian response in the potassium concentration range between 10^−5.9^ M and 10^−1^ M of K^+^ ions and may be applied in the pH range between 2 and 10.5.

## 1. Introduction

Nanomaterials, commonly known as materials with a single unit sized up to 100 nm, have been widely applied for the construction of ion-selective electrodes (ISEs) [1]. The application covered introducing them to ionophores [2], using them as sensing transducers [3], and finally making them solid-contact layers in all-solid-state electrodes [4].

All-solid-state electrodes have their origin in early 1970s when Catrall and Freiser [5] made a significant contribution to the field of ion-selective electrodes by presenting a coated-wire electrode built of platinum wire and ion-selective membrane. The novelty of this construction raised from the absence of an inner solution was an integral part of earlier types of ISEs. Twenty years later this construction of an electrode was enhanced by implementing the solid-contact layer in between electronic conductor (metal/wire electrode) and ionic conductor (ion-selective membrane), which made it possible for the charge to be easily transferred amidst materials of different types of transductions [4]. Hence, the main feature and, simultaneously, the main requirement for solid-contact layers are ion-to-electron transduction properties [6,7,8]. Ion-to-electron transducer materials are characterized either by redox or double-layer capacitance.

Nanomaterials belong to a group with large surface area, which ensures that high double-layer capacitance is formed in the interface between the solid-contact layer and membrane. The wide implementation of nanomaterials results from their unique physical and chemical properties, such as large surface/volume ratio and large pore volume, ensuring unique surface chemistry and good conductivity [9]. These features make them appropriate for being applied as solid-contact layers, as the requirements for such materials with a high surface, ensuring high electrical capacity, is essential for obtaining a stable and fast response of sensors [10].

This work describes the design of a nanomaterial composed by modification of graphene flakes (GR) by gold nanoparticles (AuNPs) and its application in ion-selective electrodes’ construction as solid-contact layer. Graphene (GR) belongs to the group of carbon nanomaterials that are among the most widely used materials for solid-contact layer. It is a two-dimensional carbon material with only one atomic layer that shows unique properties such as a fast electron transportation, a high surface area, a high thermal conductivity, and excellent mechanical properties [11,12]. The use of graphene as standalone material as well as a component of composite material was reported in literature by Li at al. [13] (standalone GR layer) and by our group in [14] (as a part of graphene-carbon black composite) and [15] (in graphene-ruthenium dioxide composite). Gold nanoparticles (AuNPs) feature excellent conductivity, high surface area, and redox properties [16] which, together, makes them excellent materials for solid-contact layers in ion-selective electrodes. As a standalone material for intermediate layer, gold nanoparticles were successfully applied into ion-selective electrodes construction by Jaworska et al. [17].

Although both materials used in this work were already applied as solid-contact layers individually, combining them into one material allowed us to obtain a hydrophobic layer, of much higher contact angle and greater electrical parameters in comparison with other, as mentioned above, graphene–based composites. To the best of our knowledge, our group is the first one to apply graphene/gold nanoparticles material in the construction of ion-selective electrodes and obtain satisfactory results demonstrating that the modification of graphene with gold allows for enhancing electrical and wetting properties of carbon nanomaterial. Obtained results are promising in the context of designing solid-contact ion-selective electrodes and were presented in the next sections. This paper has been divided into the following parts: material characteristic and electrical and analytical characterization of ion-selective electrodes with nanomaterial layer.

## 2. Materials and Methods

### 2.1. Chemicals

The solid-contact layer consisted of graphene obtained from ACS Material, gold nanoparticles, and was provided by 3D-nano, Poland, with Sodium borohydride NaBH_4_ acting as a reductor. Graphene flakes decorated with gold nanoparticles were dispersed in dimethylformamide (DMF) (POCH).

The membrane components: potassium ionophore I (Valinomycin), lipophilic salt–potassium tetrakis(4-chlorophenyl)borate (KTpClPB), 2-nitrophenyl octyl ether (o-NPOE), and poly(vinyl chloride) (PVC) were purchased from Sigma-Aldrich and dissolved in Tetrahydrofuran (Sigma Aldrich, Saint Louis, MO, USA).

Potassium chloride (KCl) was purchased from POCH (Gliwice, Poland) and solutions of K+ ions concentration from 10^−7^ to 10^−1^ M were used for potentiometry, chronopotentiometry, and EIS measurements. Hydrochloric acid and sodium hydroxide used for adjusting the pH value of solutions during the pH sensitivity test were purchased from POCH, Gliwice, Poland.

### 2.2. Preparation of SC-ISEs

Graphene modified with gold nanoparticles was implemented onto the glassy carbon disc electrode’s surface and the material was examined for its suitability as intermediate layers in ion-selective electrodes.

Solid-contact layer was casted onto glassy carbon disc electrode surface using the drop casting method, which among other techniques is considered to be the fastest and the simplest. In order to obtain the solid-contact layer, the DMF-based solution of graphene modified with nanogold was prepared by a simple one-pot method. For this purpose, HAuCl_4_ (1 mg/mL; abcr GmbH, 99.9% metal basis) was mixed thoroughly with DMF/graphene dispersion (4 mg/mL, ACS Material) for 20 min with the assistance of sonification. Afterwards, NaBH_4_ (0.1 mg/mL; Sigma Aldrich, purum p.a. ≥ 96%), acting as a reducing agent, was added rapidly to the solution being mixed under vigorous stirring. Such a prepared solution was washed by centrifugation (5000 rpm, 10 min) in order to remove the excess of the reducing agent, and subsequently redispersed in DMF. The washing procedure was repeated three times.

At the beginning of the preparation process, glassy carbon disc electrodes were polished with alumina slurries and rinsed with water and methanol, alternately. Clean and dried electrodes were casted with 20 µL of solid-contact layer solution. DMF was removed from casted layers by evaporation process until only the solid particles were left at the electrode’s surface. At this stage, the layers were tested without membrane in order to examine their electrochemical properties.

The experimental part was followed by casting the obtained layers with 60 µL of ion-selective membrane solution of the following composition: potassium ionophore I 1.10% (*w*/*w*), KTpClPB 0.25% (*w*/*w*), o-NPOE 65.65% (*w*/*w*), and PVC 33.00% (*w*/*w*). All membrane components of total weight 0.125 g were dissolved in 1 mL of THF. The solvent was evaporated in the room temperature and after 24 h, the conditioning process begun as electrodes were placed into 0.01 M KCl solution in order to saturate the membrane with K^+^ ions.

One group of electrodes was prepared separately as a control group of coated-disc electrodes obtained by casting electrodes surface directly with ion-selective membrane, without the intermediate layer in between.

Both the group of coated-disc and solid-contact electrodes consist of three replicate electrodes.

### 2.3. Methods

Amongst the methods applied in the experiment for examination of graphene/gold nanoparticles layers, Transmission Electron Microscope (TEM), contact angle microscope, Electrochemical Impedance Spectroscopy (EIS), and chronopotentiometry method were used for material characterization and the potentiometry method was implemented to evaluate the influence of the layers’ presence on the ion-selective electrodes’ analytical performance.

The microstructure of studied graphene/gold nanoparticles material was investigated with Transmission Electron Microscope Tecnai 20 X-TWIN (FEI, Hillsboro, OR, USA) fitted with Energy Dispersive X-Ray Analysis (EDAX) and High Angle Annular Dark Field (HAADF) detectors.

In order to examine the wetting properties of the obtained material, contact angle microscope Theta Lite microscope with One Attension software by Biolin Scientific (Gothenburg, Sweden) was implemented into studies.

For chronopotentiometry and electrochemical impedance spectroscopy method, glassy carbon disc electrodes were covered with the studied graphene/gold nanoparticles material and placed into a measuring cell in sequence, together with reference Ag/AgCl electrode with 3 M KCl solution (type 6.0733.100 from ΩMethrom, Herisau, Switzerland) and auxiliary-glassy carbon electrode. All electrodes were connected to the Autolab analyzer (Eco Chemie, Utrecht, The Netherlands). The cell was filled with 0.01 M KCl solution acting as an electrolyte. Both chronopotentiograms—the potentiometric response of electrode recorded with time in the forced current conditions and Nyquist plots, on which the imaginary part of impedance (Z″) is plotted on the y axis, and the real part of impedance (Z′) on the x axis, were collected using NOVA 2.1 software.

For potentiometry method all prepared ion-selective electrodes with gold nanoparticles layer and potassium-selective membrane and the coated-disc electrode were connected to the 16-channel mV-meter (Lawson Labs, Inc., Malvern, PA, USA) and measurements were conducted against Ag/AgCl electrode (type 6.0733.100 ΩMetrohm, Switzerland) reference electrode in the presence of platinum auxiliary electrode. For this measurement, KCl solutions of 10^−1^ to 10^−7^ M concentration were used as K^+^ ions standard solutions.

## 3. Results

### 3.1. Material Characteristic

The examined material was graphene modified with gold nanoparticles, which was tested using TEM microscope to explore the microstructure. Further, a contact angle microscope was used to evaluate the wetting properties and Autolab analyzer was used to evaluate the electrochemical behavior.

#### 3.1.1. Microstructure Investigation

**Figure 1:**
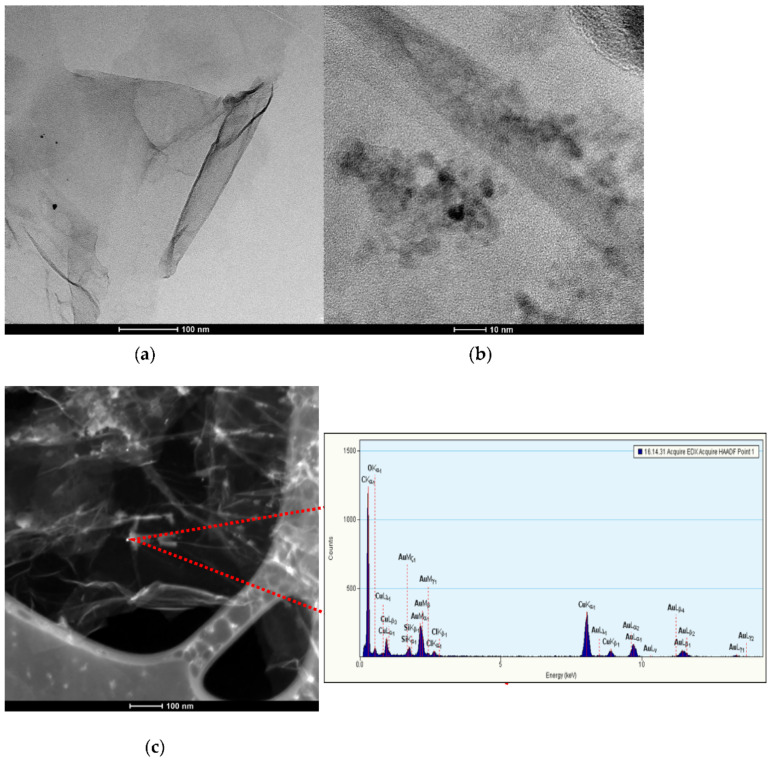
TEM (**a**) and HR-TEM (**b**) scans of graphene modified with gold nanoparticles with EDS spectrum and analysis (**c**). BF (Bright Field), HR (High Resolution), and HAADF (High-angle Annular Dark-field) detectors were applied to obtain scans a, b, and c, respectively.

Pictures obtained from the TEM microscope were presented in Figure 1. Figure 1a,b displays a microstructure of graphene modified with gold nanoparticles, from distant to very close view and the scans were collected with the use of BF (Bright Field) and HR (High Resolution) detector. The scan presented in Figure 1c was obtained with the use of HAADF detector and the point of EDS analysis is marked. Spectra corresponding to the points are presented next to the scan.

In the BF scan nanoparticles are visible as dark points on the graphene flakes (of lighter color) while in the HAADF (High-angle Annular Dark-field) scan, gold nanoparticles can be spotted as light points. This was also confirmed by the EDS analysis, as for the lightest point (indicated in the Figure 1c), the analysis showed the presence of gold. The quantitative analysis in the marked point was as follows: gold 19.44% (*w*/*w*) and carbon 80.55% (*w*/*w*).

HR-TEM scan allowed to evaluate the size of a single particle. According to the scale, the diameter of the gold nanoparticle is equal to approximately 5 nm.

All scans display single nanoparticles of gold evenly distributed on the graphene flakes without an evidence of an agglomeration process, which in the context of obtaining the material of a highly active surface area is greatly desirable.

#### 3.1.2. Contact Angle Measurements

**Figure 2:**
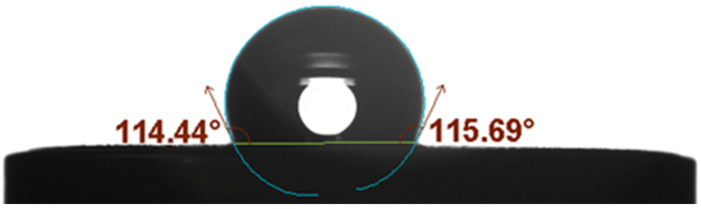
Contact angle of the studied graphene/gold nanoparticles material calculated by One Attension software.

When choosing or designing the material for solid-contact layer, wetting properties of material should be taken into consideration. The hydrophobicity of the material is highly desired as it prevents the formation of water layer on the surface of the solid-contact layer (under the membrane). The wettability of graphene/gold nanoparticles material was tested using the contact angle microscope; the water droplet of 5 µL was discharged onto the materials’ surface, while software measured the angle between the surface and the tangent to the water drop, as shown in Figure 2.

The software gave the contact angle a value of 115°, which indicates that the designed material is hydrophobic. The obtained results were compared with those presented in our previous work concerning the graphene/ruthenium dioxide material. The contact angles of 49.5° and 61.9° were reported for graphene individually and graphene/ruthenium dioxide composite, respectively [15]. Those values are considerably lower than the one obtained for graphene/gold nanoparticles material, which indicates that the addition of gold nanoparticles not only significantly affects graphene wetting properties but also makes it a great material for a solid-contact layer in ion-selective electrodes. In this case, a higher water contact angle is likely caused by the increase of the roughness of graphene after its modification with gold nanoparticles, since a rough surface can influence the boundary between a water drop and the examined surface [18,19].

Implementing hydrophobic intermediate layer into ion-selective electrodes’ construction was repeatedly shown to prevent the formation of water film. This is not only crucial in the context of analytical performance of electrodes but also when it comes to the lifetime of electrodes, as a water layer under an ion-selective membrane causes the delamination of membrane and, in consequence, mechanical damage of the electrode.

#### 3.1.3. Electrical Characteristics of the Intermediate Layer and Electrode

Designed electrodes were characterized with the use of two electrochemical techniques: chronopotentiometry and electrochemical impedance spectroscopy.

**Figure 3:**
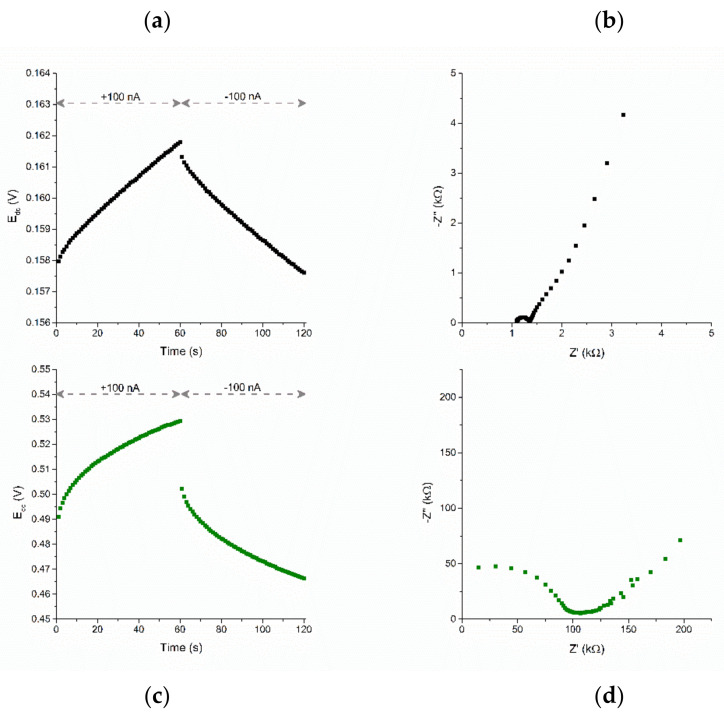
Electrochemical characteristics of the intermediate layer (**a**,**b**) and ready-to-use electrode (**c**,**d**) represented by chronopotentiograms and the Nyquist plot.

Based on the chronopotentiograms recorded with the use of electrodes covered with tested graphene-gold nanoparticles layer, the electrical parameters of the layer were evaluated including electrical capacitance and resistance. With solid-contact layers for ion-selective electrodes, the higher the electrical capacity, the more stable the potentiometric response of electrodes is. Therefore, high values of electrical capacitance and low values of resistance are considered as desirable features. Figure 3a presents a part of recorded chronopotentiograms consisting of two steps; first 60 s with +100 nA current flow and another 60 s with −100 nA. For the tested layer, the value of electrical capacitance was calculated for the linear part of the chronopotentiometric curve, which equaled to 1.80 ± 0.06 mF, indicating that the modification of graphene with gold nanoparticles allowed to almost double the capacity, in comparison to the results reported by Paczosa-Bator [14]. The determined value of the resistance equaled to 2.30 ± 0.08 kΩ.

Another method used for the intermediate layer characterization is electrochemical impedance spectroscopy. The Nyquist plot, being the visualization of the imaginary part of the impedance (−Z″) (axis y) and the real part of impedance (Z′) (axis x), is presented in the Figure 3b. The electric charge capacity of the double layer can be easily determined from the low frequency data using the C = 1/(2πf(−Z″)) relation, where f is the frequency and −Z″ is the corresponding imaginary part of the impedance. The capacitance value was determined on the basis of three frequencies 13.9, 19.3, and 26.8 mHz and was respectively 2.75, 2.58, and 2.39 mF.

After being examined, GR-AuNPs–based layers were covered with ion-selective membrane solution in order to obtain ready-to-use solid-contact ion-selective electrodes. The electrical properties of the studied electrodes were also evaluated using chronopotentiometry and electrochemical impedance spectroscopy. The dependencies obtained in the subsequent measurements are shown in Figure 3c,d.

Based on the chronopotentiogram, presented in the Figure 3c, recorded with a current flow of ±100 nA, the electric charge capacity equal to 277 ± 5 µF (for the linear part of recorded curve) and the resistance equal to 137 ± 1 kΩ were determined. Taking into consideration the results obtained for single layers and layers covered with potassium membrane, it can be seen that the presence of ion-selective membrane increases the resistance of electrodes and decreases the value of electrical capacitance.

The EIS measurement results are shown again in the Nyquist plot in Figure 3d. The shape of the graph is characteristic for solid-contact electrodes with a transducer layer with a capacitive conduction mechanism. The visible semicircle corresponds to the high range of measurement frequencies and is related to the parameters of the ion-selective membrane, such as bulk resistance or geometric capacitance. The low measuring frequency corresponds to the line visible on the chart, which corresponds to the characteristics of the processes taking place at the interface between the solution and the ion-selective membrane. On the basis of this range, we determine the electrical capacity of the double layer, according to the formula given above for solid-contact layers. Again, the capacitance value was calculated for several of the lowest frequencies: 10, 13.9, and 19.3 mHz and was equal to 224, 210, and 195 µF, respectively.

### 3.2. Potentiometric Tests

For the potentiometric tests, the studied graphene/gold nanoparticles layer was covered with ion-selective membrane, and the ready-to-use electrode was connected to the potentiometer. The characteristics of solid-contact and coated-disc electrodes were recorded in standard KCl solutions in order to determine such parameters of electrodes as standard potential and sensitivity (measured as slope of calibration curve).

#### 3.2.1. Potentiometric Response

**Figure 4:**
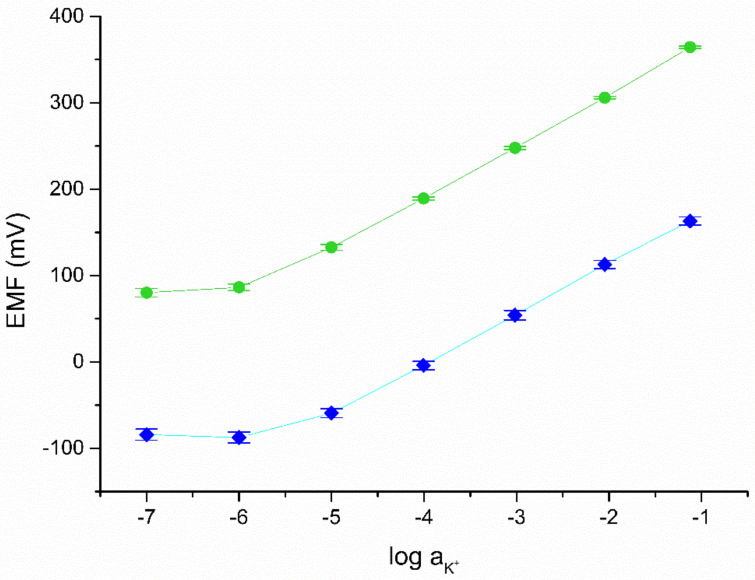
Potentiometric response of GC/GR-AuNPs/K^+^-ISE (green plot) and coated disc electrode (blue plot) after 24, 48, and 72 h of conditioning in 0.01 M KCl.

Calibration curves were recorded after 24, 48, and 72 h of conditioning in 0.01 M KCl solution for each group of electrodes (solid-contact and coated-disc) and presented in Figure 4. Standard deviations for the electrode’s parameters were calculated based on the results obtained for one electrode representing each group during the three days of conditioning process and were collected in Table 1.

All tested electrodes exhibit the slope of calibration curve consistent with the theoretical Nernstian value.

**Figure 5:**
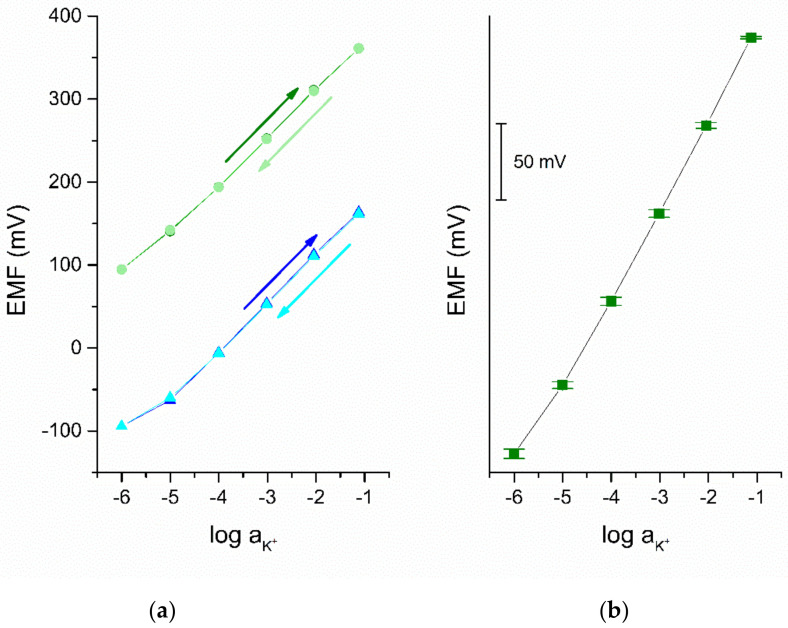
Calibration curves of (**a**) GC/GR-AuNPs/K+-ISM (olive and light green) and coated-disc electrode (blue and cyan) with arrows pointing at the increase (olive and blue arrow) or decrease (light green and cyan arrow) in the concentration of measured standard solutions and (**b**) averaged calibration obtained from three copies electrodes from the group of GC/GR-AuNPs/K^+^-ISM with standard deviation.

To compare the repeatability of the response of the electrodes with solid-contact layer and coated disc electrode, calibration curves were recorded firstly by measuring standard solutions of increasing concentration (from 10^−6^ to 10^−1^ M) (arrow pointing up) and subsequently solutions of decreasing concentration (arrow down), as shown in Figure 5a.

The reproducibility of electrodes was tested within a group of GC/GR-AuNPs/K^+^-ISM electrodes of the same composition and the convergence of the potentiometric response towards potassium ions was examined, as shown in Figure 5b. This parameter was quantified by the values of standard deviations, calculated based on the results obtained for three items prepared in an identical manner. The absolute values of standard deviation are minor and no more than 3.06 mV for the concentration of K^+^ ions equal to 10^−6^ M, which proves the great reproducibility of the tested GC/GR-AuNPs/K^+^-ISM electrodes.

#### 3.2.2. Reversibility of Response

**Figure 6:**
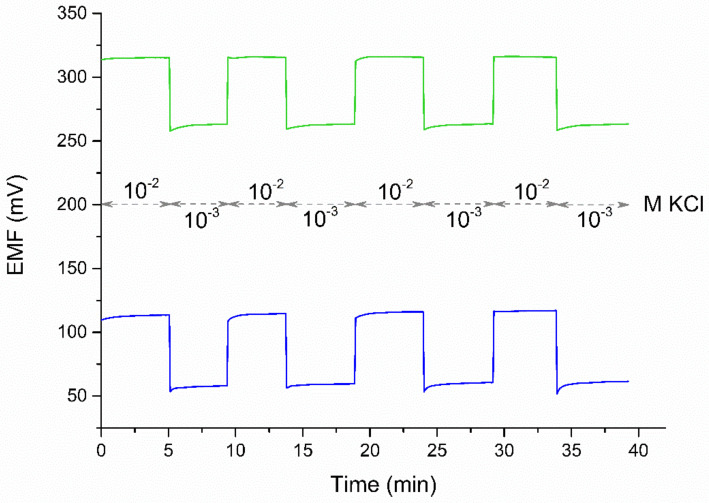
The reversibility of electrodes’ potentiometric response–GC/GR-AuNP/K^+^-ISM (green line) and coated-disc electrode (blue line).

The reversibility of the response was examined individually for the group of GC/GR-AuNPs/K^+^-ISM electrodes and the group of coated disc electrodes. The reversibility of the potential can be determined by the standard deviation of the potential value obtained for a solution of the same concentration after successive rapid changes in the concentration, as shown in Figure 6. The standard deviation of the potential for the concentration of 10^−2^ M KCl was 0.2 mV for the GC/GR-AuNPs/K^+^-ISE electrode and 1.7 mV for the coated-disc electrode. For the concentration of 10^−3^ M KCl the results corresponded to 0.1 and 1.5 mV, respectively.

#### 3.2.3. Stability of Response

**Figure 7:**
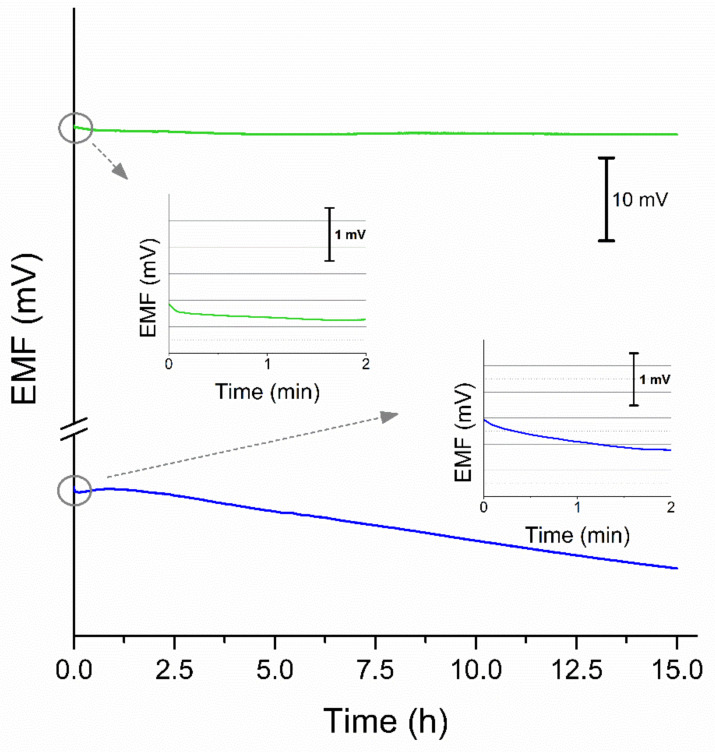
Potentiometric response of GC/GR-AuNP/K^+^-ISM (green line) and coated-disc electrode (blue line) measured over 15 h with a closer look at the first 2 min of measurement presented on the insets.

Another two important features of ion-selective electrodes are stability and response time, defined as the potential change over the time and the time needed to reach the 95% of stable potential value, respectively. Both parameters were tested during 15 h long measurement in 0.01 M K^+^ ions solution and the potential response was recorded with time, as presented in the Figure 7. The potential drift calculated during the measurement equaled to 0.036 mV/h for GC/GR-AuNPs/K^+^-ISM electrode and 0.70 mV/h for coated-disc electrode. The decrease of potential drift value observed for solid-contact electrode contrary to coated disc electrode is certainly due to the high electrical capacity of electrodes with the graphene/gold nanoparticles layer. Applying the material of high electrical capacity to the electrodes’ construction enables it to sustain the potential equilibrium in the presence of external disturbances. Electrodes with layers characterized by the high electrical capacitance parameter exhibit stable potentiometric responses over time and insensitivity to the perturbations that may occur during the measurement, which results in their great potential stability over the time of measurement.

Insets to the Figure 7 give a closer look at the first 2 min of measurement displaying the difference in response of solid-contact and coated-disc electrodes. The response of graphene/gold nanoparticles-based electrodes, after being placed in K^+^ ions solution was faster than for coated-disc electrode and the time needed to reach the stable value of potential was much shorter. For solid-contact electrodes it took only a few seconds to reach the potential value equal to 95% of equilibrium potential, while CG/K^+^-ISM electrode did not reach the stable potential value through the time of measurement and the significant drift was observed.

#### 3.2.4. Light and pH Sensitivity

The examination of potential stability in the stable conditions of potentiometric measurement was followed by the examination of potentiometric response of electrodes in varying conditions, such as light intensity fluctuation and dynamic changes in the pH value. Both tests were performed for one item from the group of studied graphene/gold nanoparticles electrodes and the light test was additionally conducted in the presence of coated-disc electrode as a control.

**Figure 8:**
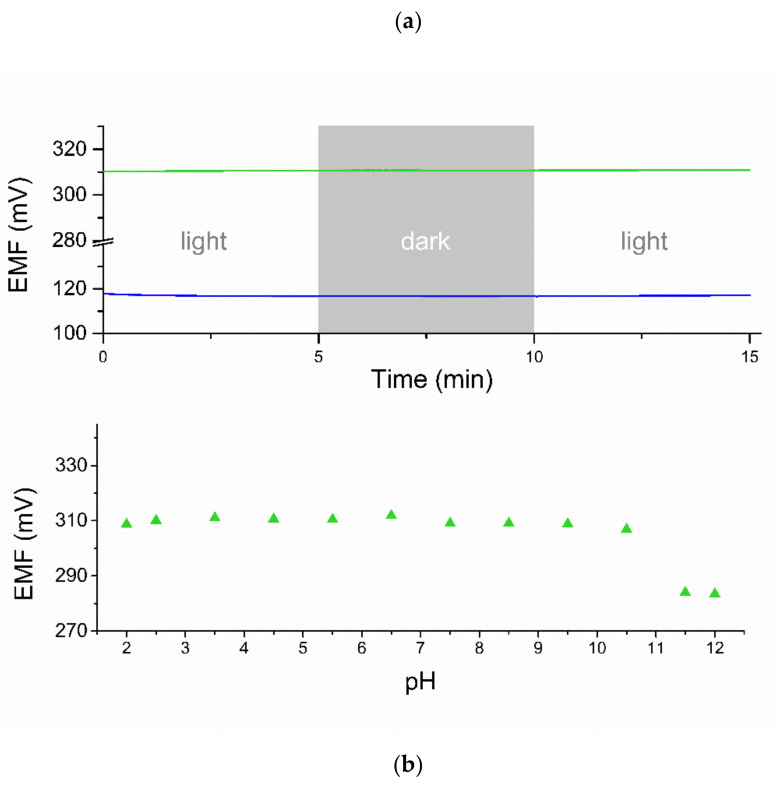
The potentiometric response of studied electrodes; GC/GR-AuNP/K^+^-ISM (green line) and GC/K^+^-ISM (blue line) electrode showing their insensitivity to (**a**) light exposition, (**b**) dynamic pH value.

Figure 8a presents the course of light sensitivity test. Both electrodes studied, solid-contact and the control coated disc electrode, were placed into K^+^ ions standard solution of 0.01 M concentration and the intensity of light was changed from day light, to complete darkness, then back to day light. The potentiometric response of electrodes with time was recorded. Visible changes in the electrodes’ response were not detected, therefore it is concluded that the studied GC/GR-AuNP/K^+^-ISM electrodes are insensitive to the changes in the light exposure and may be applied alternatively in day light and dark conditions.

With ion-selective electrodes it is important to examine the pH range in which the potential response is stable and repeatable, therefore, the range in which electrodes may be applied. For the purpose of this test 0.01 M K^+^ ions standard solutions were prepared and titrated with sodium hydroxide or hydrochloric acid in order to establish their pH value. A wide range of solutions of varying pH value (from 2 to 12) was prepared and the potentiometric response of the GC/GR-AuNP/K^+^-ISM electrode was recorded in each solution. Results of the test are presented in Figure 8b. As can be seen, studied electrode exhibit a stable potentiometric response in the solutions of pH values from 2 to 10.5 and the decrease in potential was observed for higher pH values. It can therefore be concluded that designed graphene/gold nanoparticles-contacted electrodes can be applied within the pH range of 2 to 10.5.

## 4. Conclusions

This work explores the association of two nanomaterials by combining them into one nanocomposite material by modifying the structure of graphene with nanometric particles of gold. Throughout the paper we have repeatedly proven that the properties of both materials translate into the electrodes’ performance. The modification of graphene with gold nanoparticles (size approximately 5 nm) allowed us to create a layer of rough microstructure observed with the TEM microscope a high water contact angle of 115°, ensuring the hydrophobic properties of designed solid-contact layer. This feature of solid-contact layer prevents the water layer formation, therefore protecting the electrodes from mechanical damage caused by the membrane delamination. The addition of nanometric particles to carbon material caused the increase of surface area of material and in consequence the increase of electrical capacitance value. High electrical capacitance of solid-contact layer (of 1.8 mF) translated into high electrical capacity of graphene/gold nanoparticles-contacted electrodes (equal to 280 µF). The designed electrodes are insensitive to the light conditions, exhibit stable potentiometric response (with the potential drift of only 0.036 mV/h) and may be applied in the pH range between 2 and 10.5 and potassium ions concentration range between 10^−5.9^ M to 10^−1^ M.

The paper has proved that the modification of carbon nanomaterial allows for obtaining more satisfactory results in the context of solid-contact ion-selective electrodes and presents a universal approach to designing robust potentiometric sensors for future research.

## Figures and Tables

**Table 1:** Calibration curves parameters calculated for one electrode representing each group after 24, 48, and 72 h of conditioning (n = 3).

Group of Electrodes	Sensitivity S [mV/dec]	Normal Potential E_0_ [mV]	Limit of Detection [M]	Linear Range [M]
**GC/GR-AuNPs/** **K^+^-ISM**	59.6 ± 0.2	429.2 ± 0.6	10^−5.9±0.1^	10^−5.5^–10^−1^
**GC/K^+^-ISM**	57.9 ± 0.5	224 ± 4	10^−5.5±0.1^	10^−5^–10^−1^
